# Comparative visual outcomes of EDOF intraocular lens with FLACS vs conventional phacoemulsification

**DOI:** 10.1097/j.jcrs.0000000000001027

**Published:** 2022-08-31

**Authors:** Jia Xu, Wenbo Li, Zhe Xu, Binsong Zhao, Yueyang Zhong, Kai Wang, Xin Liu, Xiaohui Song, Yinhui Yu, Yanan Zhu, Qiaomei Tang, Ke Yao

**Affiliations:** From the Eye Center of the 2nd Affiliated Hospital, School of Medicine, Zhejiang University, Hangzhou, Zhejiang, China (J. Xu, Li, Z. Xu, Zhong, Wang, Liu, Song, Yu, Zhu, Tang, Yao); Zhejiang University College of Computer Science and Technology, Hangzhou, Zhejiang, China (Zhao).

## Abstract

To investigate the visual quality after femtosecond laser–assisted cataract surgery (FLACS) and conventional phacoemulsification surgery (CPS) with the implantation of an extended depth-of-focus (EDOF) intraocular lens (IOL) TECNIS Symfony.

**Setting:**

Eye Center, the Second Affiliated Hospital of Zhejiang University, Zhejiang, China.

**Design:**

Prospective cohort study.

**Methods:**

Patients were given the option to choose FLACS or CPS and were implanted with a TECNIS Symfony. Main outcome measures were postoperative examinations that included defocus curves, contrast sensitivity (CS), optic path difference aberrometry scan, anterior segment photography, and questionnaires.

**Results:**

261 patients (261 eyes) were enrolled. The circularity index of FLACS was more precise than that of CPS (*P* = .001). FLACS demonstrated a significantly lower IOL decentration (*P* = .011) and IOL tilt (*P* = .009). FLACS presented a significantly lower total aberration (*P* < .001), higher-order aberrations (HOAs) (*P* = .001), coma (*P* = .001), and spherical aberration (*P* < .001). With IOL decentration of more than 0.40 mm, total internal aberration (*P* = .023) and HOAs (*P* = .045) were significantly deteriorated. As for defocus curve, FLACS was better at −1.00 diopter (*P* < .01). The FLACS group achieved higher CS at 6 to 18 cycles per degree under glare condition (*P* < .05). With regard to photic phenomena, the FLACS group received better outcomes (*P* < .05).

**Conclusions:**

With the implantation of an EDOF IOL, FLACS could precisely control the shape and size of the capsulotomy and induce a significantly better-centered IOL, leading to higher visual performance compared with CPS.

## Abstract 2

With the implantation of an EDOF IOL, FLACS could precisely control the shape of the capsulotomy and induce a significantly better-centered IOL, leading to higher visual performance compared with CPS.

At present, cataract surgery, which was originally only used monofocal intraocular lenses (IOLs) to restore vision at 1 specific distance, has evolved into refractive cataract surgery.^1^ The extended depth-of-focus (EDOF) IOL TECNIS Symfony (Johnson & Johnson Vision), a new concept IOL, based on a proprietary achromatic diffraction echelette design, is proven to be capable of restoring the patient's far, intermediate, and part of near vision, producing EDOF and minimal interference light phenomenon.^2,3^

The decentration and tilt of the IOL is a crucial factor influencing the visual quality of patients postoperatively.^4^ The architecture of the capsulorhexis has a great influence on the position of the lens, which in turn affects the refractive effect.^5^ If the capsulotomy is too large, the IOL optical system might be tilted or decentered, resulting in optical aberrations and damage to the retinal image.^6^ Higher-order aberrations (HOAs) can produce visual errors, such as night vision difficulties and glare, halos, and other light disturbances, which could lead to falls, postural stability, and other difficulties in activities of daily living.^7,8^ The complex optical design of an EDOF IOL showed higher sensitivity to decentration, which might lead to larger HOAs.^9–11^

In recent years, with the advent of femtosecond lasers in cataract surgery, an anterior capsulotomy can be centered on the pupil center, pupil maximized center, lens center of the scanned capsule, or custom placement, with a predictable and more regular ring-shaped capsule rupture.^12^ Although a large number of comparative studies have been conducted on femtosecond laser–assisted cataract surgery (FLACS) and conventional phacoemulsification surgery (CPS), no larger sample clinical data, implanting with an EDOF IOL, have been published yet. Thus, the aim of this study was to compare the visual outcomes and photic phenomena between FLACS and CPS in cases implanted with the TECNIS Symfony IOL.

## METHODS

### Study Design

This prospective, examiner-blinded consecutive cohort study was performed with the approval of the Institutional Review Board of the Second Affiliated Hospital of the School of Medicine, Zhejiang University, Hangzhou, China, and in accordance with the tenets of the Declaration of Helsinki. After receiving a complete explanation of the study, all patients signed the written informed consent. This trial was registered at www.chictr.org.cn (identification number ChiCTR-ONN-17010319).

### Patients

This trial included patients undergoing cataract surgery between July 2017 and March 2021 at Eye Center, Second Affiliated Hospital, College of Medicine, Zhejiang University. Inclusion criteria were aged from 45 to 85 years, no active ocular disease except cataracts, received FLACS or CPS in 1 eye, nonsevere dry eye, angle κ within 0.5 mm, no pupillary abnormality, and no surgery within 3 months. Exclusion criteria were complications during surgery, reduced zonular/capsular stability, corneal astigmatism higher than 1.5 diopters (D), previous refractive surgery, poor mobility, IOL dislocation, posterior capsule opacification, or any ocular comorbidity (amblyopia, retina, or optic nerve pathology) that may hamper postoperative acuity.

All patients had a comprehensive preoperative ophthalmologic examination that included the measurement of uncorrected distance visual acuity, optical biometry (IOL Master700, Carl Zeiss Meditec AG), slitlamp examination, tonometry (NT-510, Nidek Co., Ltd.), corneal topography by Scheimpflug imaging (Pentacam, Oculus Optikgeräte GmbH), and dilated fundoscopy.

Patients who met the above criteria were adequately informed about the nature and possible consequences of the study and the characteristics of the IOL. Patients were consecutively enrolled after signing consent forms with the exception of dropout replacements. All patients were given the option to choose FLACS or CPS with the implantation of EDOF IOL TECNIS Symfony.

### Surgical Technique

IOL power was chosen to target emmetropia ±0.5 D. Every patient accepted the standard surgical procedure. In the Femto group, disposable interface contact lenses with suction rings (SoftFit Patient Interface, Alcon LenSx, Inc.) were used for the corneal applanation. LenSx software (v. 2.23, Alcon LenSx, Inc.) was used to create a 5.0 mm capsulotomy, and nuclear prefragmentation was performed to obtain 6 pieces in a cross pattern. In the Phaco group, anterior capsules were treated conventionally (Amvisc Plus, Bausch & Lomb, Inc.). Capsule forceps were used to complete a 5.0 mm continuous curvilinear capsulorhexis.

In both groups, a 2.0 mm single-plane main incision and a 0.8 mm side-port corneal incision were made with a keratome. Phacoemulsification was performed using a standard stop-and-chop technique with the longitudinal phacoemulsification system (Stellaris, Bausch & Lomb, Inc.). After the IOL implantation, the ophthalmic viscosurgical device material was removed from the anterior chamber and the capsular bag by irrigation/aspiration. All incisions were left sutureless. All patients received standard regimen consisting of topical dexamethasone tobramycin 4 times a day for 2 weeks and pranoprofen for 1 month postoperatively.

### IOLs

The TECNIS Symfony IOL is an extended-range-of-vision IOL based on the diffractive achromatic technology. It is a single-piece, UV-filtering, open-loop haptic, hydrophobic acrylic folding IOL with an overall diameter of 13.0 mm and an optic diameter of 6.0 mm. According to the manufacturer, this IOL provides an elongated focal area, rather than 1 or more individual focal points.^13^

### Outcome and Assessment

The routine examinations for all patients were performed within 1 month preoperatively and postoperatively at 1 week, 1 month, and 3 months. The additional follow-up assessment was at 3 months postoperatively. Each measurement was performed by the same technicians who performed certain functional tests but were ignorant of the surgical approach to all operated eyes.

UDVA, slitlamp anterior segment examination, direct fundoscopy, and tonometry were examined at the postoperative routine visit. As for the additional follow-up assessment, the following parameters were evaluated: visual acuity, defocus curve, contrast sensitivity (CS), an optic path difference aberrometry scan (OPD), and anterior segment photography with a dilated pupil. Monocular uncorrected and corrected distance visual acuities were measured at far (5 m), intermediate (80 cm), and near (40 cm) distances. Distance-corrected visual acuity was measured at intermediate (80 cm) and near (40 cm) distances. In addition, a monocular defocus curve was tested from −4.00 to +2.00 D in 0.5 D increments. CS with or without glare (45 cd/m^2^) under mesopic conditions was measured using the CSV-1000 contrast test (Vectorvision, Inc.) based on best distance-corrected status and at a 2.5 m testing distance.^14^ An optic path difference aberrometry scan (OPD Scan III, Nidek Co., Ltd.) was completed at a 5.0 mm pupil diameter using tropicamide eyedrops 0.5%. The root mean square of tilt, coma, trefoil, spherical aberration, HOAs, and total aberration was calculated. AutoCAD 2020 image processing for Windows software was used to measure the area and perimeter of the capsulorhexis and the IOL centration depending on the anterior segment photography. Decentration of the IOL was defined as the distance between the pupil center and the center of the IOL. The circularity index was given by the formula: 4π (area/perimeter^2^), and a circularity index of 1 implied a perfect circle. Besides, to evaluate the effect of IOL decentration and tilt on the optical quality for the EDOF IOL, we further divided all the patients into 2 groups separately (with a decentration of <0.40 mm or ≥0.40 mm; with a tilt of <0.25 μm or ≥0.25 μm).

Also, all patients were asked to complete the Questionnaire for Visual Disturbances (QUVID) to assess and evaluate the visual symptoms postoperatively. With regard to photic phenomena, the QUVID measures the frequency, severity, and degree of bother associated with 7 different visual phenomena (eg, glare, halos, and double vision) by standard photographs. Patients were asked to score each item (0, 1, 2, 3, and 4; a higher score means worse photic phenomena) in relation to their symptoms and list other photic phenomena that bothered them.

### Statistical Analysis

Data are presented as mean ± SD. Statistics were analyzed using SPSS for Mac software and SPSS Statistics for Windows software (v. 26.0, IBM Corp.). The normality of data distribution was assessed using the Shapiro-Wilks test. Comparisons between 2 groups were made using *t* tests or Wilcoxon-Mann-Whitney *U* tests, depending on data normality and homogeneity of variance. For categorical data, chi-square tests were applied. The correlations between parameters were assessed using the Spearman rank correlation coefficient (*r*). *P* values less than 0.05 were considered statistically significant. All tests were analyzed in a 2-tailed style.

## RESULTS

A total of 261 eyes of 261 patients attended to the follow-up visit. A total of 131 eyes were treated by FLACS (the Femto group), whereas 130 eyes were treated by CPS (the Phaco group). Missing data were due to personal inconvenience, refusal to mydriasis for OPD scan, or temporary device failure. No significant difference was found between the 2 groups regarding preoperative characteristics (Supplemental Table 1, http://links.lww.com/JRS/A666). No intraoperative complication occurred.

### IOL Decentration

**Figure 1.**
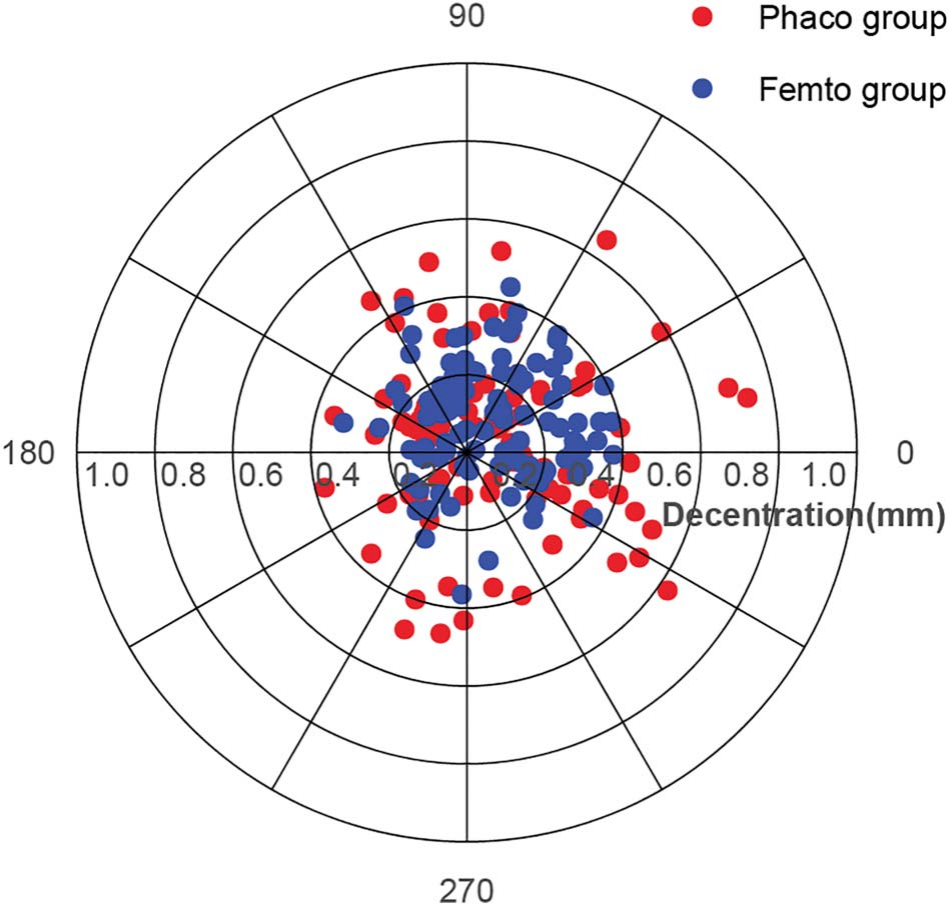
Distribution of IOL decentration in the total sample.

Postoperatively, the decentration of the IOL and the area and perimeter of the capsulorhexis were also calculated and compared between groups. As shown in Table 1, the IOL decentration was significantly smaller in the Femto group than that in the Phaco group (*P* = .011). Compared with the Phaco group, the circularity index was closer to 1 (*P* = .001), and the area and perimeter of the capsulorhexis seemed closer to 5.0 mm (*P* < .001) in the Femto group (Table 1 and Supplemental Figure 1, http://links.lww.com/JRS/A665). Also, the distribution of IOL decentration in both groups is illustrated in Figure 1.

**Table 1.** Decentration of the IOL and capsulorhexis measurements after IOL implantation

Parameter^a^	Phaco	Femto	*P* value^b^
IOL decentration (mm)	0.27 ± 0.15	0.21 ± 0.10	.011*
Circularity index	0.97 ± 0.12	0.98 ± 0.11	.001**
Capsulorhexis area (mm^2^)	21.29 ± 3.21	19.63 ± 2.00	<.001^c,^***
Capsulorhexis perimeter (mm)	16.75 ± 1.93	16.03 ± 1.61	<.001***

*P < .05, **P < .01, and ***P < .001

a Data are presented as mean ± SD

b Mann-Whitney *U* test

c *t* test

### Wavefront Aberrations

As shown in Table 2, tilt (*P* = .009), total aberration (*P* < .001), coma (*P* = .001), spherical aberration (*P* < .001), and HOAs (*P* < .001) were significantly lower in the Femto group than those in the Phaco group. No significant difference was observed in terms of trefoil aberration.

**Table 2.** Aberrations for a 5.0 mm pupil after IOL implantation (RMS)

Aberrations^a^	Phaco	Femto	*P* value^b^
Tilt	0.33 ± 0.23	0.25 ± 0.14	.009**
Total aberration	1.18 ± 0.47	0.98 ± 0.28	<.001***
Trefoil	0.26 ± 0.17	0.22 ± 0.14	.226
Coma	0.16 ± 0.12	0.11 ± 0.07	.001***
Sphere	0.19 ± 0.09	0.14 ± 0.07	<.001***
HOAs	0.19 ± 0.10	0.16 ± 0.07	.001***

RMS = root mean square

**P < .01 and ***P < .001

a Data are presented as mean (μm) ± SD

b Mann-Whitney *U* test

**Figure 2.**
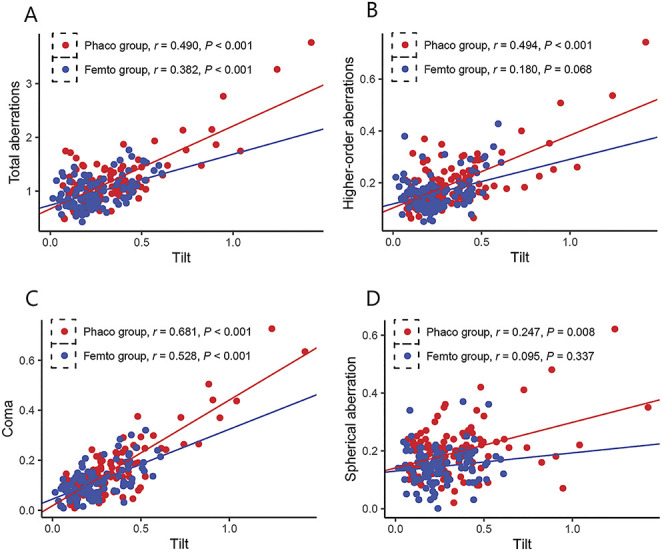
Effect of tilt on aberrations of total aberrations (*A*), HOAs (*B*), coma (*C*), and spherical aberration (*D*) with the pupil diameter of 5.0 mm.

The correlations between tilt and total aberration, HOAs, coma, and spherical aberration were analyzed (Figure 2). Total aberrations (Femto *r* = 0.382, *P* < .001; Phaco *r* = 0.490, *P* < .001), HOAs (Femto *r* = 0.180, *P* = .068; Phaco *r* = 0.494, *P* < .001), and coma (Femto *r* = 0.528, *P* < .001, Phaco *r* = 0.681, *P* < .001) were correlated with tilt in both groups. In addition, spherical aberration was correlated with tilt in the Phaco group (*r* = 0.247, *P* = .008), but not in the Femto group (*r* = 0.095, *P* = .337).

As shown in Supplemental Table 2 (http://links.lww.com/JRS/A666), with IOL decentration of more than 0.40 mm, total aberration (*P* = .023) and HOAs (*P* = .045) were significantly deteriorated. No significant difference was observed in terms of coma, trefoil, or spherical aberration.

The Femto group exceeded in MTF values at 5 and from 35 to 60 cycles per degree (cpd) for 5.0 mm pupil compared with the Phaco group (Supplemental Figure 2, http://links.lww.com/JRS/A665). The Strehl ratio was also higher in the Femto group than that in the Phaco group for 5.0 mm (0.02 ± 0.01 vs 0.02 ± 0.02, *P* = .048) pupil.

### Visual Acuities and Refractive Outcomes

Postoperatively, there were no significant differences between uncorrected and corrected visual acuity at far, intermediate, and near distances, along with spherical equivalents (SE) (Supplemental Table 3, http://links.lww.com/JRS/A666).

### Defocus Curve

**Figure 3.**
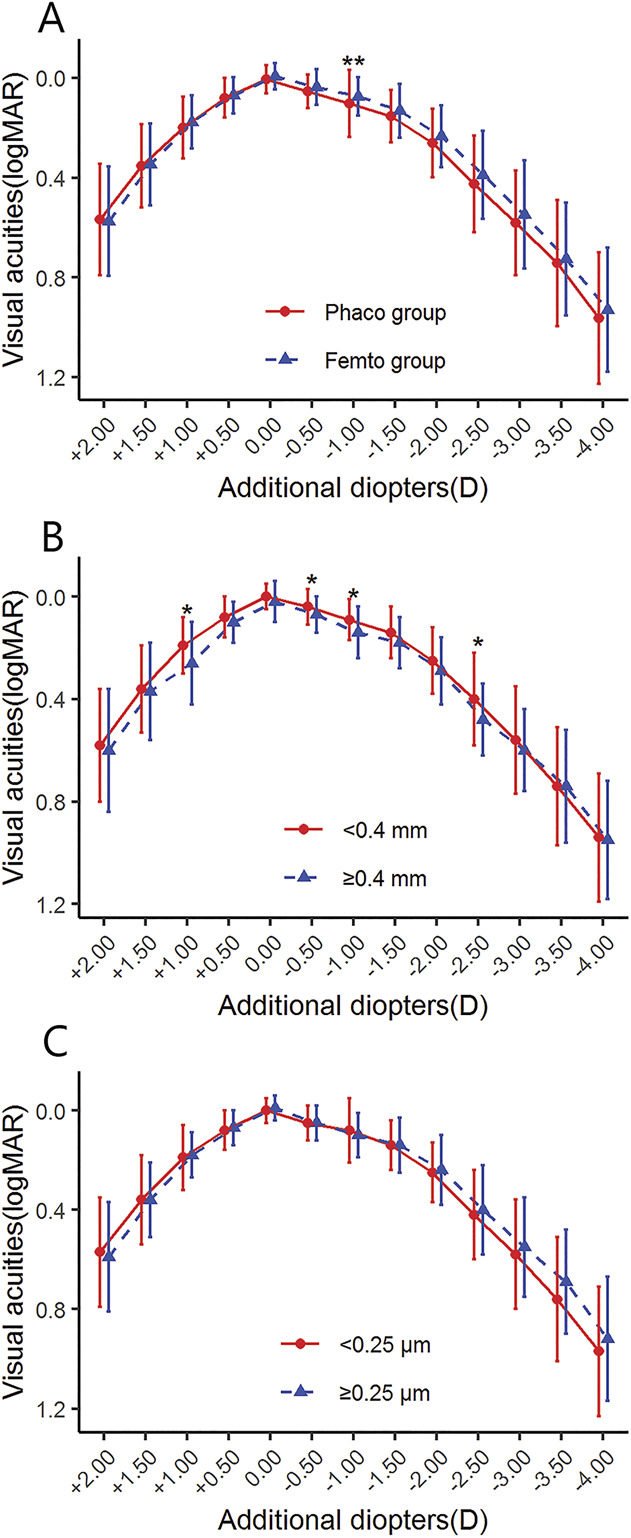
Defocus curves after IOL implantation between the Femto and the Phaco groups (*A*) and the groups divided separately with IOL decentration (*B*) or tilt (*C*). <0.4 mm = IOL decentration <0.40 mm; ≥0.4 mm = IOL decentration ≥0.40 mm; <0.25 μm = tilt <0.25 μm; ≥0.25 μm = tilt ≥0.25 μm. **P < .01.

As shown in Figure 3, the defocus curve of the 2 groups was equality smooth, and the values revealed no significant differences, except that the Femto group performed better at −1.00 D (*P* < .01) compared with the Phaco group. The defocus curve of the group with IOL decentration of more than 0.40 mm lagged at +1.00 D, −0.50 D, −1.00 D, and −2.50 D (*P* < .05). The values revealed no significant differences between the 2 groups divided with a tilt of <0.25 μm or ≥0.25 μm.

### Contrast Sensitivity

**Figure 4.**
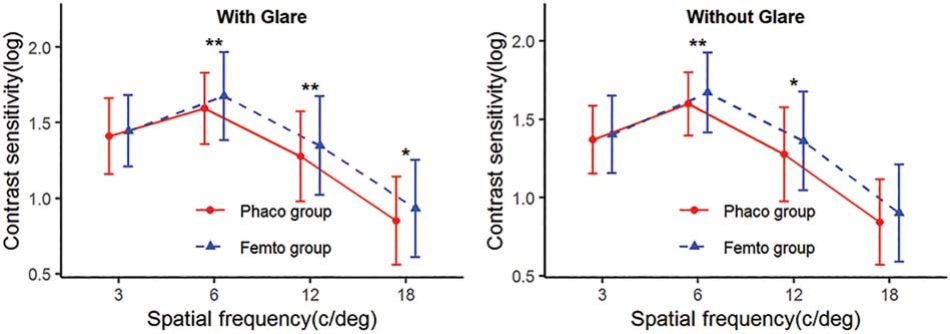
Contrast sensitivities with and without glare under mesopic condition after IOL implantation. *P < .05; **P < .01.

Compared with the Femto group, CS was significantly reduced at 6 to 18 cpd spatial frequency in the presence of glare and at 6 to 12 cpd spatial frequency without glare in the Phaco group (Figure 4).

### Questionnaire Evaluation

As for visual disturbances, eyes in the Phaco group were more likely to suffer from starburst. Also, they seemed to suffer more in the frequency, severity, or level of bother with halo and hazy, blurred, double vision (Supplemental Table 4, http://links.lww.com/JRS/A666). Other 15 symptoms the patients mentioned were all related to dry eyes, such as photophobia, eye pain, and excessive discharge.

## DISCUSSION

Since the invention of phacoemulsification, cataract surgery technology has undergone continuous development. The main purpose of cataract surgery today is not only to focus on distance vision of the naked eye but, more importantly, to pay attention to the postoperative visual quality and comfort and satisfaction of patients. However, concerned with the high economic costs, whether femtosecond surgery brings any additional clinical benefits for patients is questioned.^15–17^ The use of EDOF IOLs has been plagued with reduced CS, halos, and a variety of visual complaints.^18–20^ It was reported that many of these intolerances were actually attributable to uncorrected astigmatism and optical imperfections that might be overcome with the use of FLACS, although no study had yet reported the relationship between visual quality and FLACS or CPS.^21^ To the authors' knowledge, this study is the first prospective study comparing clinical outcomes between FLACS and CPS with the implantation of EDOF IOL TECNIS Symfony.

In this study, clinical outcomes at 3 months after operation, including IOL decentration, wavefront aberrations, visual acuities, refractive outcomes, defocus curve, CS, and questionnaire, were evaluated and compared between the Femto and Phaco groups. We showed that FLACS could make the capsular bag well encapsulate the IOL, inducing a better-centered IOL position. Previous study has investigated the effects of capsulorhexis size and shape on the tilt and decentration of IOLs that a larger capsulorhexis size with bad coverage of the IOL edge is significantly correlated with IOL decentration.^22^ In this study, the circularity index of FLACS was more precise than that of CPS, and the area and perimeter was more stable, which indicates a better-sized capsulotomy. The data of IOL decentration verify that a more precise capsulotomy in the femtosecond case leads to a better-centered IOL.

To analyze the visual quality, wavefront aberration analysis isolates the effects of lower-order aberrations (defocus and astigmatism) and HOAs, which is overwhelmingly affected by the anterior corneal surface and crystalline lens.^23^ Consistent with previous data, we observed significantly smaller tilt, total aberration, coma, and HOAs in the femtosecond group at a 5.0 mm pupil.^24–27^ Zhong et al. showed similar results that no significant results were found in terms of trefoil, but the spherical aberration in the Femto group was significantly lower.^28^ Based on the 6th order Zernike polynomial decomposition, the root mean square of single second-order tilt is a prismatic error rather than a true optical aberration, only causing a shift in its position without inducing distortion of the image, and may further induce refractive error or visual defect.^29^ Significant positive correlations between tilt and total aberrations, HOAs, and coma were showed in both groups, and that total internal aberration and HOAs were significantly deteriorated with a larger decentration, matching the previous results. As for MTF values and Strehl ratios, they stand for the overall combination of corneal and intraocular optical aberrations, and objectively measure the overall optical quality of the human eye, showed that femtosecond laser capsulotomy resulted in higher MTF values and Strehl ratios, agreeing with previous study.^9^ To conclude, with regard to our statistics, off-centered and tilted IOLs can induce HOAs, which could lead to impaired optical quality.

Most published studies comparing the refractive effects of FLACS with CPS found that there was no statistically significant difference between the 2 surgical methods.^15,30–32^ However, Ruiz-Alcocer et al. found that decentration and tilt could shift the peaks of vision of the EDOF IOL, impairing visual acuity and visual quality.^11^ In our work, no significant differences were found in visual acuity or refractive outcome between the Femto and Phaco groups. As for defocus curve, on the other hand, FLACS performed better at −1.00 D. In addition, as shown in Figure 3, it can be observed that the visual acuity of the group with a greater IOL decentration was worse than that of the group with a smaller IOL decentration from +1.00 to −4.00 D, although no significant difference was found at some diopters. Our results suggested that, for the EDOF IOL, excessive decentration could adversely affect continuous visual acuity in patients.

Higher-order aberrations have a destructive effect on the optical transfer function. Decreased contrast is one such effect. Fujikado et al. found that higher-order aberrations were significantly correlated with CS at intermediate to high spatial frequencies in eyes with cataract.^33^ Furthermore, Khan et al. believed that higher-order aberrations have negative effects on all spatial frequencies.^8,34,35^ In this study, the Femto group achieved higher CS at intermediate to high spatial frequencies (6 to 18 cpd), especially at 6 to 12 cpd spatial frequency under glare condition, which indicated that patients who accepted traditional phacoemulsification were more likely to suffer from starburst, glare, halo, light spots, streaks, or color blocks in the field of vision, and the visual contrast would be worse with the appearance of strong light (such as sunlight and street lights).^5^

We applied the QUVID to investigate the visual symptoms postoperatively and observed that, compared with the Femto group, either the probability of the appearance of starburst or the frequency, severity, or level of bother with halo, hazy, blurred, or double vision was significantly higher in the Phaco group, as expected. These annoying visual phenomena are the main factors affecting the quality of life of patients. The results of this subjective questionnaire corroborated our previous analysis and verified the clinical significance of those objective visual qualities, including IOL decentration, tilt, OPD, and CS.

Our work revealed that when implanted with an EDOF IOL, the better capsulotomy provided by the femtosecond technology could ensure a more accurate position of the IOL, leading to reduced IOL decentration and tilt, lower HOAs, better functional vision, higher CS, and greater visual performance, and yield an improved life quality eventually. The combination of femtosecond technology and EDOF IOLs could better meet the needs of patients for higher life quality and might make revolutionary progress in refractive cataract surgery.

However, limitations existed in our study. First, it was not a randomized clinical trial, because of patient compliance and the relatively high cost of femtosecond surgery. Second, we only included TECNIS Symfony IOL, and therefore, the clinical outcomes after FLACS and CPS with the implantation of other types of extended-range-of-vision IOLs could not be investigated.

In summary, by investigating the visual and optical quality after FLACS and CPS with the implantation of EDOF IOL TECNIS Symfony, our study provides evidence that FLACS could precisely control the shape and size of the capsulotomy and induce a significantly better-centered IOL, leading to higher visual performance and less photic phenomena.

**WHAT WAS KNOWN**

- A better IOL position, which is mostly depending on the better-sized capsulorhexis performed using the femtosecond laser, could reduce the decentration or tilt of the IOL, resulting in the decrease in aberrations.
- Dysphotopsia symptoms, especially halos and starbursts, were reported after the implantation of EDOF IOL TECNIS Symfony.

**WHAT THIS PAPER ADDS**

- When implanted with an EDOF IOL, the better capsulotomy provided by the femtosecond technology might lead to reduced IOL decentration and tilt, lower higher-order aberrations, better functional vision, and higher contrast sensitivity and yield greater visual performance eventually.
